# Functionalized Graphene Oxide Mediated Adriamycin Delivery and miR-21 Gene Silencing to Overcome Tumor Multidrug Resistance *In Vitro*

**DOI:** 10.1371/journal.pone.0060034

**Published:** 2013-03-20

**Authors:** Feng Zhi, Haifeng Dong, Xuefeng Jia, Wenjie Guo, Huiting Lu, Yilin Yang, Huangxian Ju, Xueji Zhang, Yiqiao Hu

**Affiliations:** 1 State Key Laboratory of Pharmaceutical Biotechnology, School of Life Sciences, Nanjing University, Nanjing, P.R. China; 2 Research Center for Bioengineering and Sensing Technology, University of Science and Technology Beijing, Beijing, P.R. China; 3 State Key Laboratory of Analytical Chemistry for Life Science, Department of Chemistry, Nanjing University, Nanjing, P.R. China; 4 Modern Medical Research Center, Third Affiliated Hospital of Soochow University, Changzhou, P.R. China; Argonne National Laboratory, United States of America

## Abstract

Multidrug resistance (MDR) is a major impediment to successful cancer chemotherapy. Co-delivery of novel MDR-reversing agents and anticancer drugs to cancer cells holds great promise for cancer treatment. MicroRNA-21 (miR-21) overexpression is associated with the development and progression of MDR in breast cancer, and it is emerging as a novel and promising MDR-reversing target. In this study, a multifunctional nanocomplex, composed of polyethylenimine (PEI)/poly(sodium 4-styrenesulfonates) (PSS)/graphene oxide (GO) and termed PPG, was prepared using the layer-by-layer assembly method to evaluate the reversal effects of PPG as a carrier for adriamycin (ADR) along with miR-21 targeted siRNA (anti-miR-21) in cancer drug resistance. ADR was firstly loaded onto the PPG surface (PPG_ADR_) by physical mixing and anti-miR-21 was sequentially loaded onto PPG_ADR_ through electric absorption to form ^anti-miR-21^PPG_ADR_. Cell experiments showed that PPG significantly enhanced the accumulation of ADR in MCF-7/ADR cells (an ADR resistant breast cancer cell line) and exhibited much higher cytotoxicity than free ADR, suggesting that PPG could effectively reverse ADR resistance of MCF-7/ADR. Furthermore, the enhanced therapeutic efficacy of PPG could be correlated with effective silencing of miR-21 and with increased accumulation of ADR in drug-resistant tumor cells. The endocytosis study confirmed that PPG could effectively carry drug molecules into cells via the caveolae and clathrin-mediated endocytosis pathways. These results suggest that this PPG could be a potential and efficient non-viral vector for reversing MDR, and the strategy of combining anticancer drugs with miRNA therapy to overcome MDR could be an attractive approach in cancer treatment.

## Introduction

Multidrug resistance (MDR) is a significant obstacle for successful breast cancer chemotherapy Traditional chemotherapy or a single therapeutic strategy often fails to achieve expected results in cancer treatment due to MDR. MDR is often mediated by drug efflux transporters such as P-glycoprotein (P-gp, encoded by ABCB1), which are often overexpressed in cancer cells [1], [2]. The co-delivery of MDR-reversing agents and anticancer drugs is a promising way to overcome MDR in cancer chemotherapy [3], [4], [5], [6], [7]. Various MDR-reversing agents have been explored to enhance the efficiency of chemotherapy [8]. However, due to high inherent toxicity and resulting alterations in the pharmacokinetics of anticancer drugs, these MDR-reversing agents have very limited clinical potential [9]. MicroRNAs (miRNAs, or miRs) are a group of small non-coding RNAs (approximately 22 nucleotides), that regulate the expression of their target genes by degrading target mRNA transcripts or inhibiting target mRNA translation [10]. Distinct miRNA expression patterns are associated with various cancers and anticancer drug resistance [11]. miR-21 is overexpressed in many cancers, and its overexpression is significantly correlated with drug resistance in breast cancer [12], [13], [14]. The inhibition of miR-21 by small interfering RNA against miR-21 (anti-miR-21) can overcome multidrug resistance and restore the chemosensitivity of anticancer drugs in tumor cells [14], [15]. Thus, targeting special miRNAs opens a new avenue for the treatment of drug resistant cancers [16]. The combination of anticancer drugs with miRNA-silencing gene therapy through an effective nanocarrier system is an attractive approach to overcome MDR [1], [6], [17], [18].

**Figure 1:**
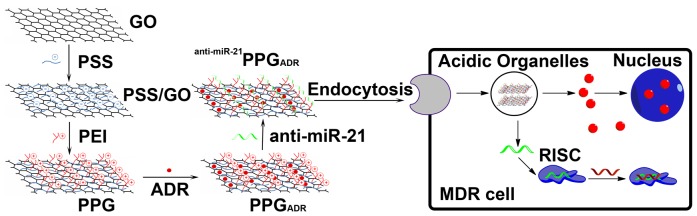
Schematic of the PPG fabrication and MDR reversion.

Graphene, a type of two-dimensional nanomaterial, has been extensively studied for its excellent physical, chemical and mechanical properties [19]. Recently, its biomedical application has emerged as an interesting field. It is often prepared as nanoelectronics, biosensors and nanocomposites. PEGylated nanoscale graphene oxide (GO) was formulated as a nanocarrier to load anticancer drugs, such as adriamycin (ADR) and SN38 [1], [20], [21]. High-efficiency loading and controlled release of ADR by GO was also achieved via π-π stacking between the drug and GO [22]. Functionalized nanoscale GO was also able to deliver oligonucleotides into cells and to protect oligonucleotides from enzymatic cleavage [23]. PEI conjugated GO as a gene delivery carrier was reported from other groups [24], [25]. Moreover, enhanced chemotherapy efficacy was achieved by sequential delivery of siRNA and anticancer drugs using PEI-grafted GO [26]. However, the combination of miRNA therapy and anticancer drugs by simultaneous delivery of siRNA and anticancer drug into cells to overcome MDR by a functionalized GO generated using the layer-by-layer assembly method as a carrier remains unexplored. As illustrated in Fig. 1, in this study, a multifunctional nanocomplex, composed of polyethylenimine (PEI)/poly (sodium 4-styrenesulfonates) (PSS)/graphene oxide (GO) and termed PPG, was successfully prepared through a layer-by-layer chemical assembly method. The efficacy of ADR-loaded PPG nanosystem (PPG_ADR_), anti-miR-21-loaded PPG nanosystem (^anti-miR-21^PPG), and ADR, anti-miR-21 co-loaded PPG nanosystem (^anti-miR-21^PPG_ADR_) on MCF-7 breast cancer cells and ADR resistant MCF-7 (MCF-7/ADR) cells was systematically investigated. Moreover, the reversal mechanism was also preliminarily investigated based on the gene inhibition, cellular uptake and endocytosis mechanism study.

## Results

### Fabrication and Characterization of PPG

**Figure 2:**
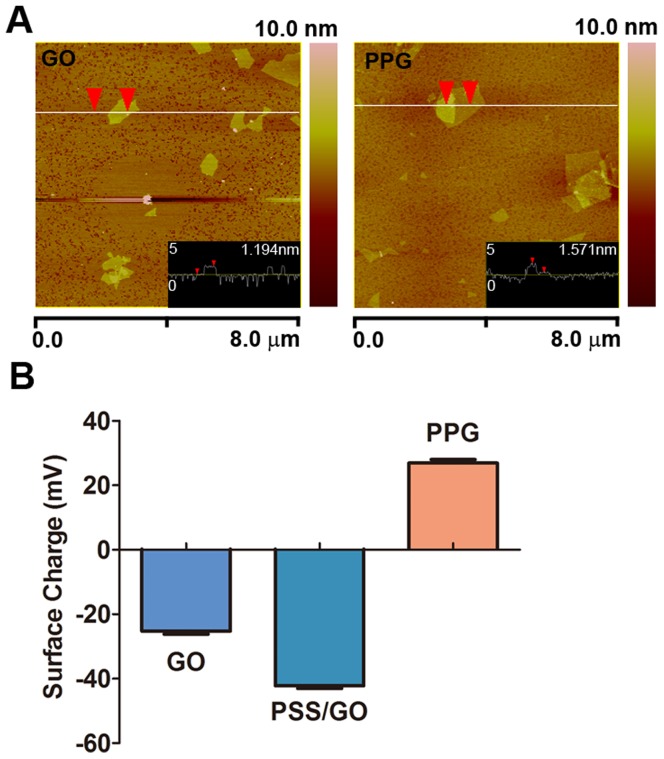
Fabrication and characterization of PPG. (A) AFM images of GO (left) and PPG (right). (B) Zeta-potential analysis of GO, PSS/GO and PPG.

The thickness of the prepared GO was about 1.2 nm, and the size distribution was within a narrow range from 50 to 300 nm (Fig. 2A left), which was in agreement with previous reports [27]. After PSS and PEI were assembled onto the GO using the layer-by-layer assembly method, many surface protuberances were observed on the surface of PPG, indicating that a large amount of PSS and PEI was immobilized onto the GO sheet (Fig. 2A right). The size of the final carrier PPG was 500±45 nm determined by AFM. The assembly process of PPG was confirmed by zeta-potential analysis. The surface zeta-potentials of GO, PSS/GO and PPG were -27.2±1.3 mV, -42.2±0.8 mV and 26.6±0.4 mV, respectively (Fig. 2B). The assemble process of PPG was also characterized by FT-IR and NMR analyses. As shown in Fig. S1 and Fig. S2, the spectrum of the PPG presented the characteristic peaks of GO, PSS and PEI, which indicated the successful assembly of the PPG. The concentration of PPG was 0.4 mg/ml as determined by TGA.

### Fabrication of PPG_ADR_, ^anti-miR-21^PPG and ^anti-miR-21^PPG_ADR_

**Figure 3:**
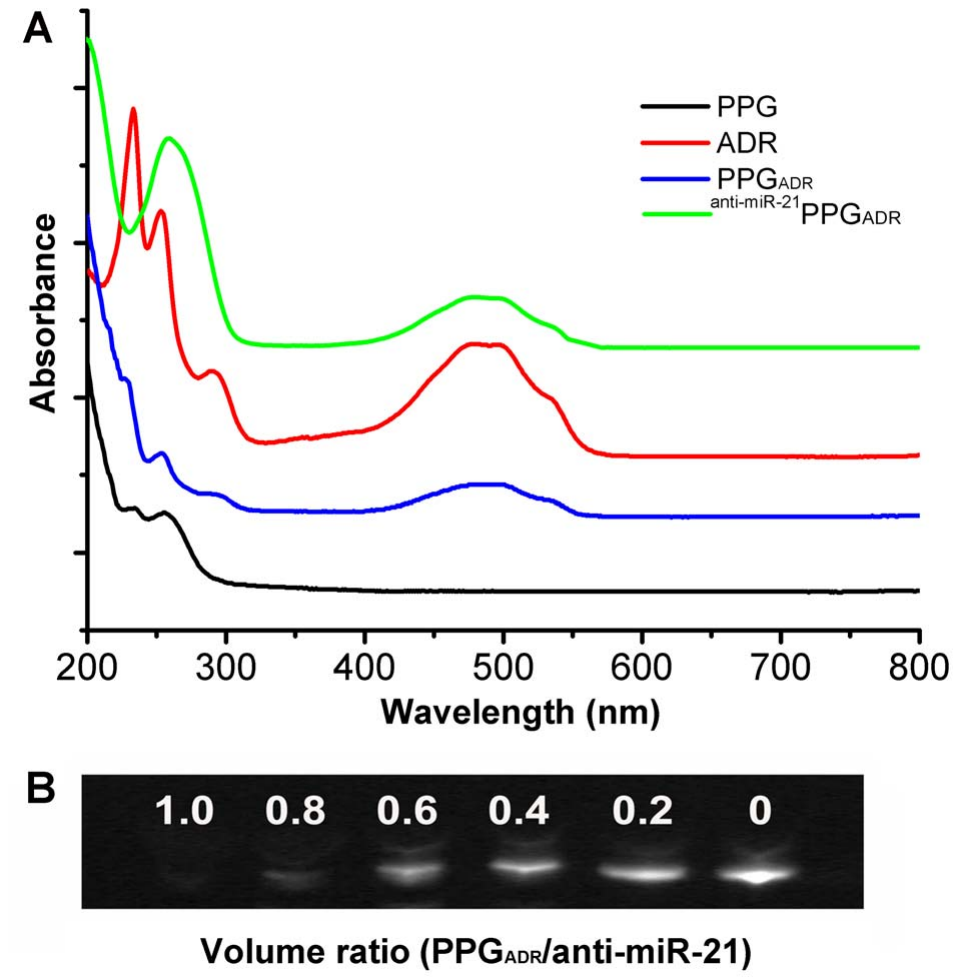
Fabrication of PPG_ADR_, ^anti-miR-21^PPG and ^anti-miR-21^PPG_ADR_. (A) UV-Vis spectra of ADR, PPG, PPG_ADR_ and ^anti-miR-21^PPG_ADR_ in aqueous solution; (B) Electrophoretic mobility of anti-miR-21 with PPG_ADR_ at different volume ratios.

Ultraviolet-visible (UV-vis) absorption spectroscopy was employed to conform the loading of ADR onto PPG. As shown in Fig. 3A, ADR exhibited a strong absorption peak at 480 nm, while PPG barely had any absorption peak after 300 nm. The spectrum of the PPG_ADR_ showed the characteristic absorption peak of ADR clearly at 480 nm, indicating the successful formation of PPG_ADR_. The amount of ADR loaded on PPG was determined by HPLC which was 0.7 mg/ml. anti-miR-21 was loaded onto PPG_ADR_ through static interaction to form ^anti-miR-21^PPG_ADR_, as described in the methods section. ^anti-miR-21^PPG_ADR_ had two strong absorption peaks at the range of 200 nm to 800 nm, one was at 480 nm which was the typical peak of ADR, and the other one was at 260 nm which indicated the binding of anti-miR-21 onto PPG_ADR_. The ability of PPG_ADR_ to form complexes with anti-miR-21 was further investigated by using a gel retardation assay. 500 pmol anti-miR-21 (100 µM, 5 µl) was added into different volumes of the PPG_ADR_ solution. The results showed that significant interaction with anti-miR-21 was achieved starting from a volume ratio of 0.8, and complete complexation was observed at a volume ratio of 1.0 (Fig. 3B). Thus, the volume ratio of 1.0 was chosen in all subsequent experiments. ^anti-miR-21^PPG was prepared by adding anti-miR-21 into PPG at the same volume ratio as used in preparation of ^anti-miR-21^PPG_ADR_.

### Co-delivery of ADR and anti-miR-21 by PPG into Cancer Cells

**Figure 4:**
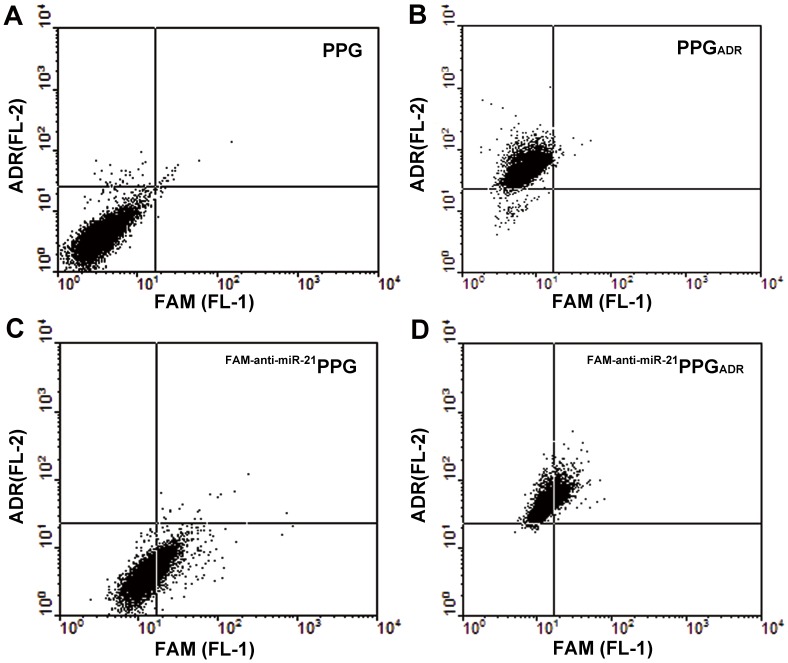
Characterization by flow cytometry of PPG co-delivering anti-miR-21 and ADR. MCF-7/ADR cells were incubated with PPG_ADR_, ^FAM-anti-miR-21^PPG, ^FAM-anti-miR-21^PPG_ADR_ at 37°C for 4 h and harvested for flow cytometry analysis. Cells treated with blank PPG served as a control.

The ability of a carrier to efficiently deliver both siRNA and anticancer drugs into cells is of particular interest for combinational cancer therapy. Herein, the co-delivery of anti-miR-21 and ADR into MCF-7/ADR cells by PPG was investigated. The presence of red fluorescence from ADR in the cells indicated that ADR was successfully delivered into cancer cells by PPG (Fig. 4B), while the green fluorescence in the cytoplasm indicated that PPG could also deliver FAM-labeled anti-miR-21 into MCF-7/ADR cells (Fig. 4C). Both green and red positive cells were observed after the cells were treated with ^FAM-anti-miR-21^PPG_ADR_ (Fig. 4D), suggesting that the PPG was able to simultaneously deliver ADR and anti-miR-21 into MCF-7/ADR cells, resulting in the co-localization of chemotherapeutic and gene therapy agents in the same cancer cells.

### Enhanced Cytotoxicity of MCF-7/ADR Cells Induced by ^anti-miR-21^PPG_ADR_

**Figure 5:**
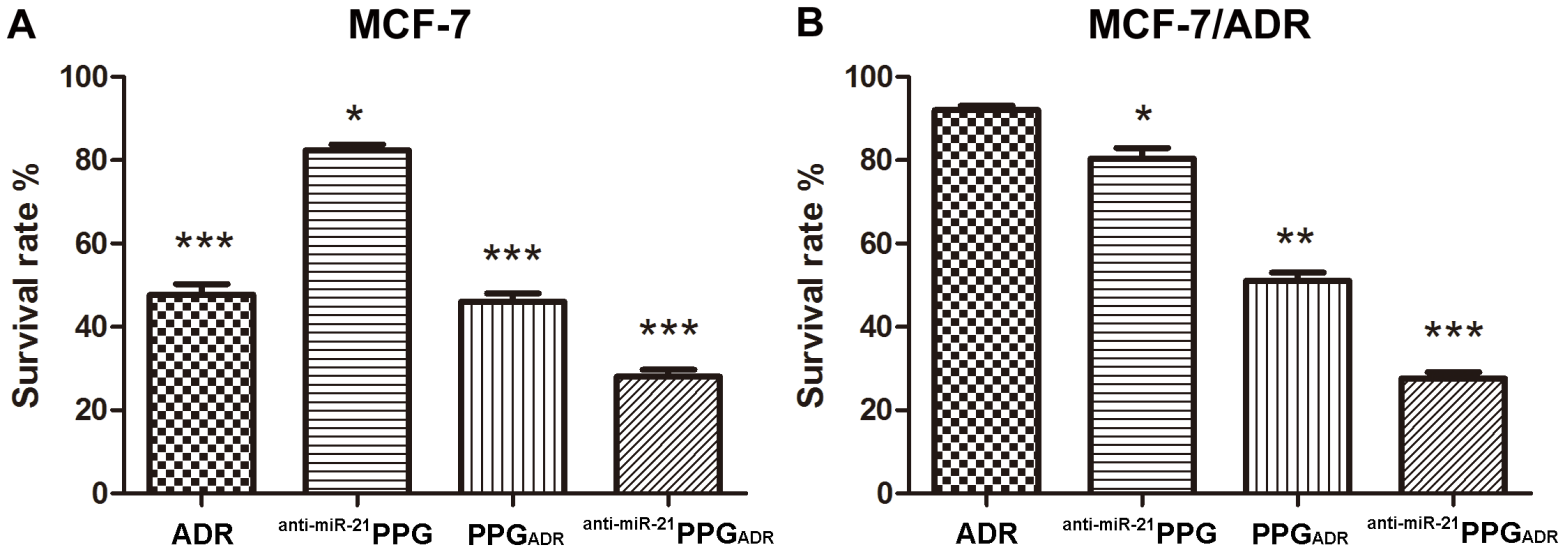
*In vitro* cellular cytotoxicity. Relative survival rate of MCF-7 cells (A) and MCF-7/ADR cells (B) after being treated with ADR, ^anti-miR-21^PPG, PPG_ADR_ and ^anti-miR-21^PPG_ADR_ for 24 h. Untreated MCF-7 and MCF-7/ADR cells were served as control in each experiment. Data are means ± SD for three separate experiments, *P<0.05, **P<0.01, ***P<0.001.

The cytotoxicities of ADR, ^anti-miR-21^PPG, PPG_ADR_ and ^anti-miR-21^PPG_ADR_ were estimated in MCF-7 (ADR sensitive) and MCF-7/ADR (ADR resistant) cells using MTT assay, and the cells were cultured with the drug treatments for 24 h. As indicated in Fig. 5, the cytotoxicity of free ADR on MCF-7/ADR was much lower than that on MCF-7 which was due to the drug resistance of MCF-7/ADR cells. The ^anti-miR-21^PPG slightly reduced cell survival rate both on MCF-7 and MCF-7/ADR cells because of the reason that miR-21 siRNA could inhibit the cell proliferation rate in cancer cells. The cell survival rate of MCF-7 cells was 35% after treated by PPG_ADR_ which was similar to the rate after treatment by free ADR, however, the cell survival rate of MCF-7/ADR cells was significantly reduced to 52% after treated by PPG_ADR_. Compared with PPG_ADR_ treatment, ^anti-miR-21^PPG_ADR_ treatment reduced the cell survival rate from 35% to 28% in MCF-7 cells and from 52% to 30% in MCF-7/ADR cells. The above results indicated that ^anti-miR-21^PPG_ADR_ effectively reversed the drug resistance of MCF-7/ADR cells.

### *In vitro* miR-21 and ABCB1 Downregulation

**Figure 6:**
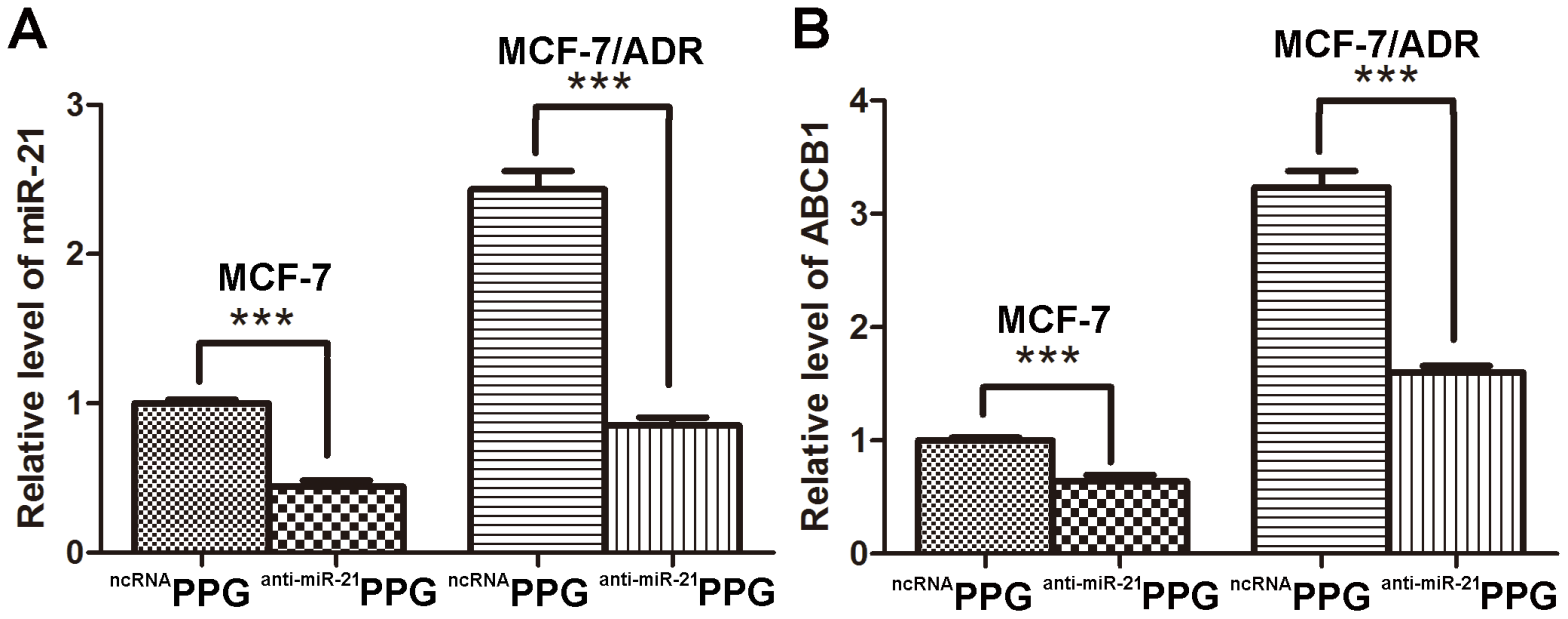
*In vitro* miR-21 and ABCB1 expression. Real time PCR analysis of relative miR-21 expression (A) and ABCB1 expression (B) in MCF-7 and MCF-7/ADR cells treated with ^ncRNA^PPG and ^anti-miR-21^PPG. The expression of miR-21 and ABCB1 in MCF-7 cells treated with ^ncRNA^PPG were arbitrarily set as 1. Data are means ± SD for three separate experiments, ***P<0.001.

The co-delivery of anti-miR-21 and ADR was expected to enhance the anticancer activity of ADR through efficient inhibition of miR-21 expression and efficient delivery of ADR, leading to resensitization of MDR cells to ADR. qRT-PCR assay was performed to detect the expression of miR-21 in MCF-7 and MCF-7/ADR cells after treatment by ^ncRNA^PPG and ^anti-miR-21^PPG for 24 h. As shown in Fig. 6A, the relative level of miR-21 expression in MCF-7/ADR cell line was about 2.5 fold higher than that in MCF-7 cell line which suggested the important role of miR-21 overexpression in MDR in breast cancer. The miR-21 expression level in MCF-7 cells and in drug-resistant MCF-7/ADR cells were reduced by 40% and 35% respectively after the cells were treated by ^anti-miR-21^PPG compared with negative control indicating that PPG could efficiently deliver anti-miR-21 into cancer cells and inhibit the expression of miR-21 (Fig. 6A). The relative expression of ABCB1 in MCF-7/ADR cell line was about 3.3 fold higher than that in MCF-7 cell line and its expression level in MCF-7 cells and MCF-7/ADR cells were reduced by 30% and 45% respectively after the cells were treated with ^anti-miR-21^PPG indicating that silencing of miR-21 could downregulate ABCB1 thus could help overcome MDR (Fig. 6B).

### ADR Accumulation and Uptake Mechanism in MCF-7/ADR Cells

**Figure 7:**
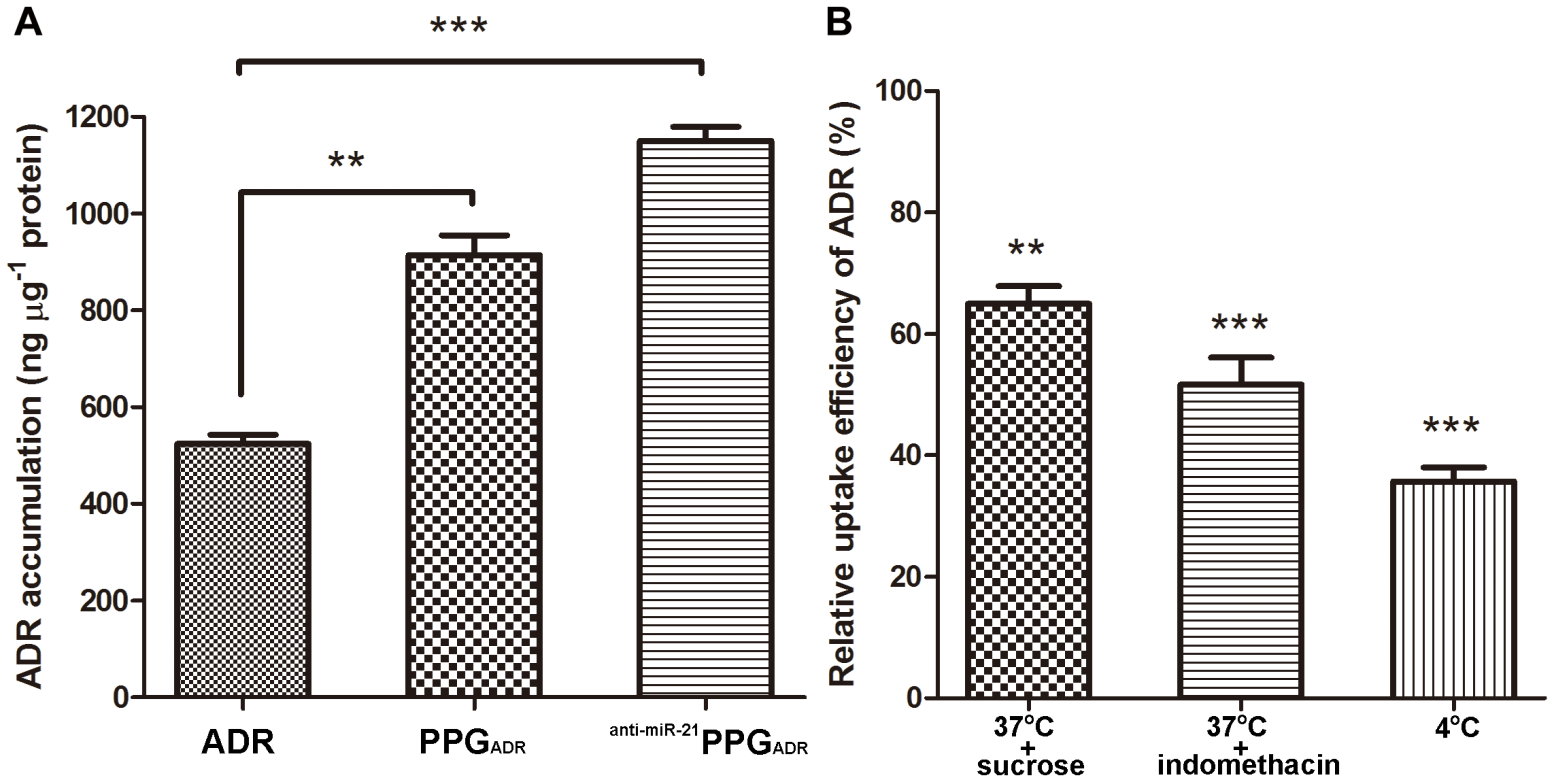
ADR accumulation and uptake mechanism in MCF-7/ADR cells. (A) ADR accumulation in MCF-7/ADR cells incubated with ADR, PPG_ADR_ or ^anti-miR-21^PPG_ADR_ for 24 h. The ADR concentration was determined by HPLC and normalized to total cell protein. Data are shown as the mean ± SD, n = 3. (B) Cellular uptake efficiency of ADR in MCF-7/ADR incubation with ^anti-miR-21^PPG_ADR_ at 37°C after treated with sucrose, at 37°C after treated with indomethacin and at 4°C. The uptake efficiency of ADR in MCF-7/ADR incubation with ^anti-miR-21^PPG_ADR_ at 37°C without any treatment was set as control. Data are means ± SD for three separate experiments, **P<0.01, ***P<0.001.

To further confirm the mechanism of enhanced cytotoxicity observed with the combination therapy, the effect of PPG on ADR accumulation in MCF-7/ADR cells was evaluated. As shown in Fig. 7A, ADR accumulation in MCF-7/ADR cells treated by PPG_ADR_ was 1.25-fold higher than that of cells treated with free ADR, implying that P-gp mediated drug efflux was partially diminished by the introduction of PPG. The inclusion of anti-miR-21 along with ADR significantly further increased ADR accumulation to a level about 2.1-fold greater than that observed with ADR solution and 1.68-fold higher than PPG_ADR_, indicating that the ability of ^anti-miR-21^PPG_ADR_ to overcome MDR was not only due to its ability to partially counteract drug transporter efflux on the cell membrane but also mediated by the reduction in miR-21 and ABCB1 expression brought about by anti-miR-21. This combined therapy was able to overcome multidrug resistance effects and restore the chemosensitivity of anticancer drugs in the tumor cells. The uptake mechanism of ^anti-miR-21^PPG_ADR_ was further investigated by examining the effects of temperature and endocytosis inhibitors on cellular uptake (Fig. 7B). The cellular uptake efficiency of ADR was obviously decreased at 4°C, while the cellular uptake efficiency of ADR at 37°C was significantly inhibited by sucrose (a clathrin-mediated endocytosis inhibitor) and indomethacin (a caveolae-mediated endocytosis inhibitor) pretreatments, which demonstrated that both caveolae and clathrin-mediated endocytosis pathways are involved in the internalization of PPG.

## Discussion

Chemotherapy is still the first line treatment in many cancers, but the potential effects of many chemotherapeutic agents are undermined by the presence of multiple drug resistance. The development of MDR can be attributed to a reduction in drug concentrations, the activation of DNA repair mechanisms and the inactivation of apoptosis pathways [28]. The combination of two or more therapeutic approaches with different therapeutic mechanisms to overcome multidrug resistance has been proposed as a means of improving the efficacy of currently available chemotherapeutic agents [5]. As a focus of in materials research, graphene has been identified as a promising candidate for potential biomedical application due to its small size, high biocompatibility and versatility of surface functionalization. Various nanomaterials closely related to graphene have been developed for biological sensing and biomedical imaging, as well as drug and gene delivery [29], [30], [31], [32]. In this study, we chose functionalized graphene oxided to overcome MDR *in vitro*.

Rather than using a functional inhibitor compound, drug resistance can potentially be overcome by targeting newly discovered small non-coding miRNA [33]. Tumor chemoresistance to certain types of drugs may be influenced by miRNA regulation. The inhibition of certain miRNAs could increase the cancer cell cytotoxicity induced by chemotherapeutics. Inhibition of miR-21 with 5-fluorouracil significanly attenuated the cell growth in glioma and in colorectal cancer [34], [35]. On the other hand, the restoration of tumor suppressor miRNAs whose expression are usually downregulated in a MDR cancer cell line compared with their parental cell line can also increase the sensitivity to several cancer chemotherapeutic drugs. For example, miR-15 and miR-16, which are negative regulators of BCL2, sensitized drug resistant cell line SGC7901/Vincristine to Vincristine-induced apoptosis [36].

Advances in nanotechnology have emerged as a new platform in controlled drug delivery and novel combination strategies. Nanoscale particles such as liposomes, polymeric micelles, dendrimers, and mesoporous silica particles between 10 and 200 nm in diameters have been used to carry broad classes of therapeutics including cytotoxic agents, chemosensitizers and small interference RNA (siRNA) [37]. These drug-loaded nanoparticles could prolong systemic circulation lifetime, sustain drug release and increase tumor drug accumulation [38].http://www.sciencedirect.com/science/article/pii/S0006295212000330 - bib0025#bib0025 anti-COX2 siRNA and dexamethasone were co-loaded onto PLGA nanoparticles to treat rheumatoid arthritis [39]. A mPEG-PLGA-b-PLL copolymer was prepared for adriamycin and siRNA delivery [40]. Effective delivery of anti-miRNAs was achieved by using functionalized gold nanoparticles which further supported the future use of miRNA in gene therapy by nanoparticles [41]. As in the multidrug resistance research field, nanotechnology also has its unique role. Many nanoparticle formulations have been developed to overcome MDR *in vitro* and *in vivo* through co-delivering combinations of chemosensitizing agents and chemotherapy agents [28]. Chemosensitizers are a group of small molecules which could inhibit drug-efflux pumps or restore the proper apoptotic signaling such as BCL-2 and nuclear factor kappa B (NF-κB) [42], [43]. In addition, the emergence of siRNA has opened a new avenue in the treatment of MDR by silencing MDR-related genes. http://www.sciencedirect.com/science/article/pii/S0006295212000330 - bib0150#bib0150Co-delivery of P-glycoprotein siRNA and doxorubicin into HeLa cells was achieved by quantum dots for reversal of multidrug resistance [44]. PLGA nanoparticles were developed to codeliver paclitaxel and Stat3 siRNA to overcome cellular resistance in lung cancer cells [45]. Magnetic Fe_3_O_4_ nanoparticle was copolymerized by ADR and MDR1 short hairpin RNA expression vector to reverse multidrug resistance in leukemia cells [46]. In this study, a novel nanocomplex was prepared via noncovalent absorption of the ADR and anti-miR-21 onto PPG which could keep the structures and properties of ADR and anti-miR-21, and could facilitate the release of these agents in cells [20]. The functionalized graphene oxide was able to co-deliver miR-21 targeted siRNA and ADR into cancer cells to overcome MDR *in vitro* which further supported the use of multifunctional nanoformulations in the treatment of MDR. Furthermore, the use of nanocarriers to deliver ADR could decrease the toxicity and side effects of ADR [47].

Small molecule drugs that enter cells through either passive diffusion or membrane translocators are susceptible to transmembrane multidrug transporter and are rapidly pumped out before they can take effect. However, nanoparticles can partially bypass the efflux pumps as they are internalized in an endocytosis-based pathway which may be mediated by clathrin, caveolae, macropinocytosis, or phagocytosis [48], [49]. After nanoparticles being engulfed by membrane proteins, these drug molecules are released far away from the membrane-bound drug efflux pumps and therefore are easy to reach and interact with their targets. Thus nanoparticle endocytic transport is a viable strategy to circumvent multidrug transporter mediated MDR effects. In our study, the uptake mechanism of PPG was also investigated in MCF-7/ADR cells. The results demonstrated that the uptake of PPG might be through energy-dependent endocytosis processes by clathrin- and caveolae-mediated pathways because the cellular uptake efficiency of PPG was significantly inhibited by low temperature, sucrose (clathrin-mediated endocytosis inhibitor) and indomethacin (caveolae-mediated endocytosis inhibitor).

### Conclusions

In summary, this study investigated the reversal of drug resistance in MCF-7/ADR cells using PPG as the carrier for ADR and miR-21 siRNA. PPG showed great superiority in co-delivering miR-21 siRNA and ADR to cancer cells, and effective miR-21 silencing and enhanced ADR accumulation were achieved. Furthermore, the uptake of PPG might be through energy-dependent endocytosis processes by clathrin- and caveolae-mediated pathways. The results suggested that use of PPG as a carrier of chemotherapeutic drugs and siRNA is favorable for the treatment of drug resistant cancers, and further investigations are still needed in future work.

## Materials and Methods

### Materials

Poly (sodium 4-styrenesulfonate) (PSS) and polyethylenimine (PEI, 25K), 3-(4, 5-dimethylthiazol-2-yl)-2, 5-diphenyl tetrazolium bromide (MTT), dimethylsulfoxide (DMSO), penicillin/streptomycin solution, sucrose and indomethacin were obtained from Sigma (Sigma-Aldrich, ST. Louis, MO, USA). Adriamycin (ADR) was obtained from Calbiochem (Calbiochem, China). RPMI-1640, fetal bovine serum (FBS) and 0.25% trypsin/EDTA were purchased from Gibco (Gibco-BRL, Gaithersburg, MD, USA). Opti-MEM was purchased from Invitrogen (Invitrogen, Carlsbad, CA, USA). Small interference RNA against miR-21 (anti-miR-21) and FAM-labeled anti-miR-21 (FAM-anti-miR-21) were supplied by Shanghai GenePharma Co. Ltd. (Shanghai, China). The primers for real-time PCR were synthesized by Sangon Biological Engineering Technology & Co. Ltd (Shanghai, China).

### Fabrication and Characterization of PPG

GO was synthesized from graphitic powder according to Hummer’s method with some modifications [27], [50]. 2 mL of GO (4 mg/mL) was mixed with 2 mL PSS solution (1 mg/mL), and the mixture was sonicated for 30 min. The suspension was then centrifuged at 15,000 g for 1 h to remove large GO and impurities, and excess PSS was removed via filtration through a Millipore Microcon 50 KDa MWCO Amicon filters (Millipore, Billerica, MA, USA). PEI (0.9 mg diluted in 1 ml water) was then added into the resulting PSS/GO solution and the concentration of PEI was controlled at 10 mM. The mixture was then sonicated for another 30 min. The final product PEI/PSS/GO termed PPG was obtained by centrifugation and filtration following the same procedure as described above. PSS was required to form the PPG nanocomplex for the introduction of PSS could increase the interlayer distance of GO and form PSS-intercalated GO which had a higher structural stability than the pristine GO due to the high melting point of PSS [51].The concentration of PPG was calculated by thermo gravimetric analysis (TGA) recorded on a Pyris TGA (PerKinElme, USA). Atomic force microscopic (AFM, Agilent 5500, U.S.A.) and Zeta-potential (Nano-Z, malvern, United Kingdom), FT-IR spectra (Nicolet 400 Fourier transform infrared spectrometer, Madison, WI) and NMR spectra (Bruker AVANCE^II^ 600 NMR) for ^1^H-NMR and ^13^C-NMR analyses were employed to characterize the assembly process of PPG.

### Fabrication of PPG_ADR_, ^anti-miR-21^PPG and ^anti-miR-21^PPG_ADR_

ADR was loaded onto PPG, denoted as PPG_ADR_, by adding ADR (5 mg/mL, dissolved in DMSO) to the PPG aqueous suspension, and the mixture was sonicated at room temperature for 0.5 h. The unloaded ADR molecules were removed by centrifugation filtration through 50 kDa MWCO Amicon filters (Millipore, Billerica, MA, USA) and washed away with water until there was no noticeable color in the filtrate solution. The product was characterized by ultraviolet-visible (UV-vis) absorption spectroscopy, as recorded with a SHIMADZU UV2450 spectrometer (SHIMADZU, Japan). The loaded ADR concentration was determined by HPLC (LC-20A, SHIMADZU, Japan; Agilent Eclipse XDB-C18 reverse phase column, USA). The sample solution was injected through a 20 µL sample loop, and a mixture of acetonitrile and KH_2_PO_4_ (0.02 mol/L) (v/v = 25/75) was used as the mobile phase at a flow rate of 1.0 mL/min. The column temperature was maintained at 25°C, and the column effluent was detected at 254 nm by a UV detector (SPD-20A, SHIMADZU, Japan). ^anti-miR-21^PPG was simply obtained by gently mixing desired amount of RNA solution and PPG aqueous solution together for 0.5 h at room temperature, while ^anti-miR-21^PPG_ADR_ was prepared by adding desired amount of RNA solution into PPG_ADR_ and mixing for 0.5 h at room temperature. The amount of anti-miR-21 solution loaded onto PPG was the same as that loaded onto PPG_ADR_. A gel retardation assay was employed to investigate the loading of anti-miR-21 onto the PPG_ADR_. Briefly, constant quantity of the anti-miR-21 was added into different volumes of PPG_ADR_ with gentle pipetting. The nanocomplex was allowed to stand at room temperature for 0.5 h before use. Then the nanocomplex was gently mixed with RNA loading buffer (Takara, Dalian, China) and was electrophoresed on a Urea-PAGE gel (20% PAGE with 7M Urea) in 1× TBE buffer at 110 mV for 0.5 h. The gel was analyzed on a gel documentation system (ChemiDoc XRS, Bio-rad, USA) to study the extent of anti-miR-21 complexation.

### Cell Culture

The ADR resistant breast cell line MCF-7/ADR and its parent cell line MCF-7 were purchased from Nanjing KeyGen Biotech. Ltd. Co. (China). The cells were cultured in RPMI-1640 medium containing 10% fetal bovine serum (FBS) and 1% penicilline/streptomycin at 37°C, in a 5% humidity atmosphere containing 5% CO_2_. The cells were subcultured routinely using trypsin/EDTA digestion upon reaching 80%–90% confluence. For the maintenance of the MDR phenotype, 1000 ng/mL ADR was added to the medium and was removed two weeks before the experimental use of the cells.

### Co-delivery of ADR and anti-miR-21 by PPG into Cancer Cells

Dual-color flow cytometry was used to characterize the PPG co-delivering ADR and anti-miR-21. The MCF-7/ADR cells (1×10^5^ cells/well) were seeded in a 6-well plate until 70% confluence was reached. ^FAM-anti-miR-21^PPG_ADR_ was prepared the same as ^anti-miR-21^PPG_ADR_. PPG, PPG_ADR_, ^FAM-anti-miR-21^PPG and ^FAM-anti-miR-21^PPG_ADR_ were added and incubated with MCF-7/ADR cells for 4 h at 37°C. Then the cells were rinsed with PBS for three times, trypsinized, collected and resuspended in 500 µl PBS. The samples were analyzed on a FACSCalibur (BD, USA) using FL1 band-pass emission for the green FAM and FL2 band-pass emission for the red ADR. Cells treated with PPG only were used as a control.

### *In vitro* Cytotoxicity in MCF-7/ADR and MCF-7 Cells

After the cells were seeded in 96-well plates at a seeding density of 1×10^4^ cells/well, they were treated with ADR, ^anti-miR-21^PPG, PPG_ADR_ or ^anti-miR-21^PPG_ADR_. Untreated cells were used as controls. The final concentrations of anti-miR-21, PPG and ADR were kept at 0.25 µM, 1 µg/mL and 1.75 µg/mL. PPG did not show any obvious cellular cytotoxity until 0.1 mg/ml in the preliminary study (data not shown). After 24 h, the treatments were removed, and fresh growth medium was added. MTT reagent (5 mg/ml) was then added to each well and incubated for a further 4 h. The culture medium was removed and DMSO (150 µl) was added. The plate was shaken for 20 s and the absorbance measured immediately at 570 nm using an ELX800 absorbance microplate reader (Bio-tek EPOCH, Winooski, VT, USA).

### *In vitro* miR-21 and ABCB1 Expression Assay

MCF-7 and MCF-7/ADR cells (1×10^5^ cells/well) were seeded in a 6-well culture plate until 70% confluence was reached. Then the cells were incubated with fresh medium containing negative control RNA (ncRNA) loaded PPG termed ^ncRNA^PPG and ^anti-miR-21^PPG for 24 h. The concentration of PPG and RNA were 1 µg/ml and 0.25 µM respectively. The cellular levels of mature miR-21 were then assessed using quantitative real-time PCR (qRT-PCR). The total RNA was extracted from cells using TRIzol reagent (Invitrogen, CA). The expression of mature miR-21 was detected by the SYBR Green miRNA assay and normalized using the 2^−*ΔCt*^ method relative to human U6. Briefly, 1 µg of total RNA was reverse-transcribed to cDNA using AMV reverse transcriptase (TaKaRa, Dalian, China) and looped antisense primers. miRNA cDNAs were generated by incubating the mixture at 16°C for 15 min, 42°C for 60 min and 85°C for 5 min. Real-time PCR was then performed on Applied Biosystems 7500 according to the standardized protocol. In each assay, 1 µl of cDNA was used for amplification. The reactions were incubated in a 96-well optical plate at 95°C for 5 min, followed by 40 cycles consisting of a 15 s interval at 95°C and a 1 min interval at 60°C. The expression of ABCB1 was detected by the SYBR Green assay and normalized using the 2^−*ΔCt*^ method relative to human GAPDH. The reverse transcription was conducted with 2 µg of total RNA using AMV reverse transcriptase (TaKaRa, Dalian, China) and real-time PCR was performed using Applied Biosystems 7500. The primers are available upon request. All reactions were performed in triplicate.

### ADR Accumulation and Uptake Mechanism in MCF-7/ADR Cells

The MCF-7/ADR cells were seeded in 6-well plates at a density of 1×10^5^ cells/well until 70% confluency, at which point they were incubated with ADR, PPG_ADR_ and ^anti-miR-21^PPG_ADR_ for 24 h. The cells were then washed thrice with ice-cold PBS, lysed in 100 µL MilliQ water and homogenized using an Omni Sonic Ruptor 250 (Omni, USA). 20 µL of the homogenate was processed for HPLC analysis. The ADR concentration in the cell lysates was determined using a standard curve and was normalized to the cell protein concentration, as determined using a Pierce protein assay kit. To investigate the underlying endocytotic mechanism that was responsible for the internalization of ^anti-miR-21^PPG_ADR_, uptake inhibition experiments were carried out with MCF-7/ADR cells. The cells were treated with 0.4 M sucrose or 100 µM indomethacin at 4°C, prior to incubation with ^anti-miR-21^PPG_ADR_. The relative uptake efficiency of ADR uptake was calculated by comparing the ADR concentration in cell lysates of different treatment groups to the ADR in the lysates of untreated cells.

### Statistical Analysis

All experiments were run in triplicate, and the data are expressed as the mean ± SE. Statistical significance was determined using Student’s t-test. Significant differences between values are designated as follows: *P<0.05, **P<0.01 and ***P<0.001.

## Supporting Information

Figure S1 **FT-IR spectra of GO, PSS, PEI and PPG.**
(TIF)

Figure S2 **Characterization of PPG by ^1^H and ^13^C MAS NMR spectra.** Liquid-state ^1^H MAS NMR spectra of GO (A), PSS (B), PEI (C) and PPG (D), Liquid-state ^13^C MAS NMR spectra of GO (E), PSS (F), PEI (G) and PPG (H).
(TIF)
