# Supplementary figures and images for: Functionalized Graphene Oxide Mediated Adriamycin Delivery and miR-21 Gene Silencing to Overcome Tumor Multidrug Resistance In Vitro
Source: PLoS One. 2013 Mar 20;8(3):e60034. doi: 10.1371/journal.pone.0060034 (PMC3603917; doi:10.1371/journal.pone.0060034)

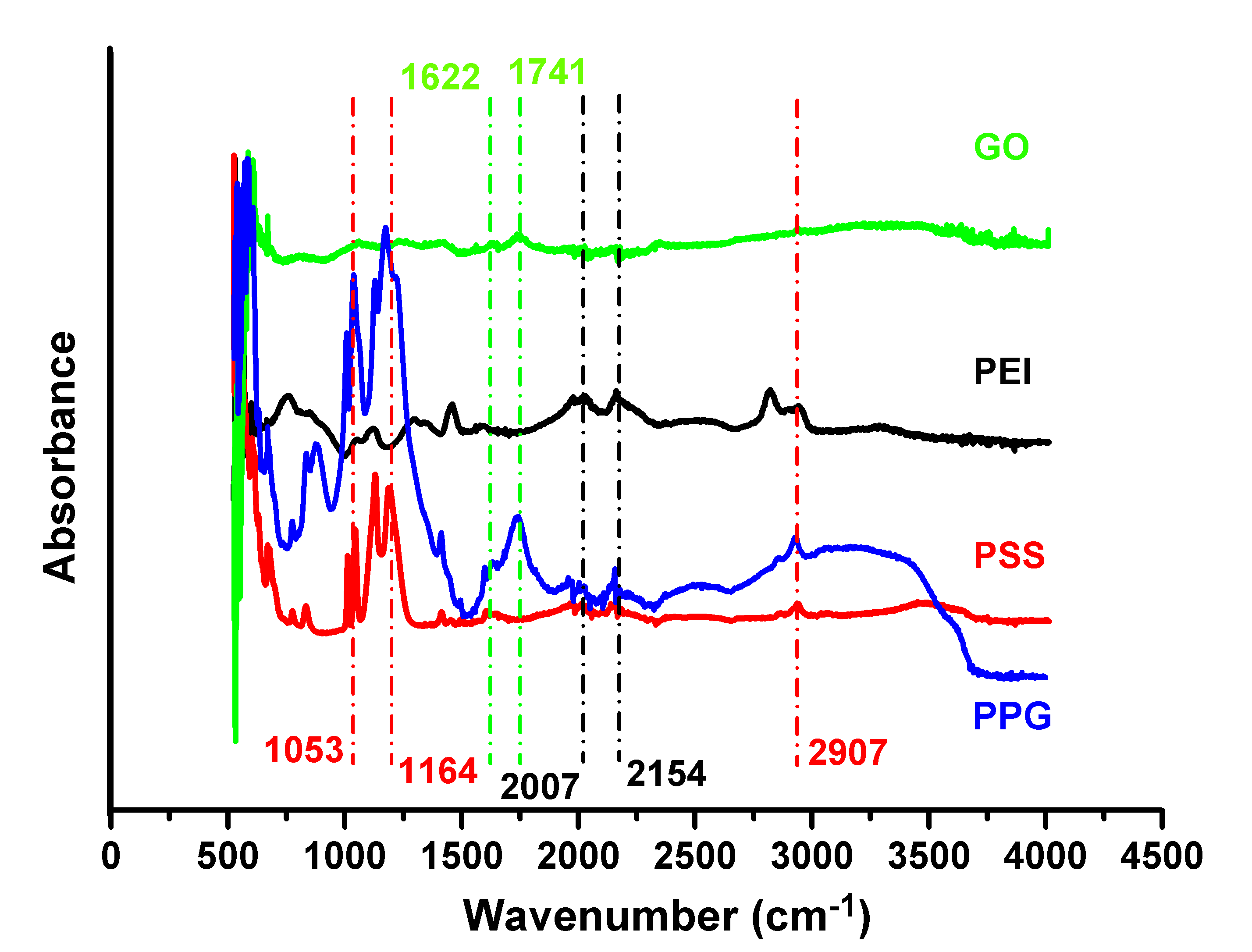

Supplement: Figure S1 — FT-IR spectra of GO, PSS, PEI and PPG. (TIF) [file pone.0060034.s001.tif]

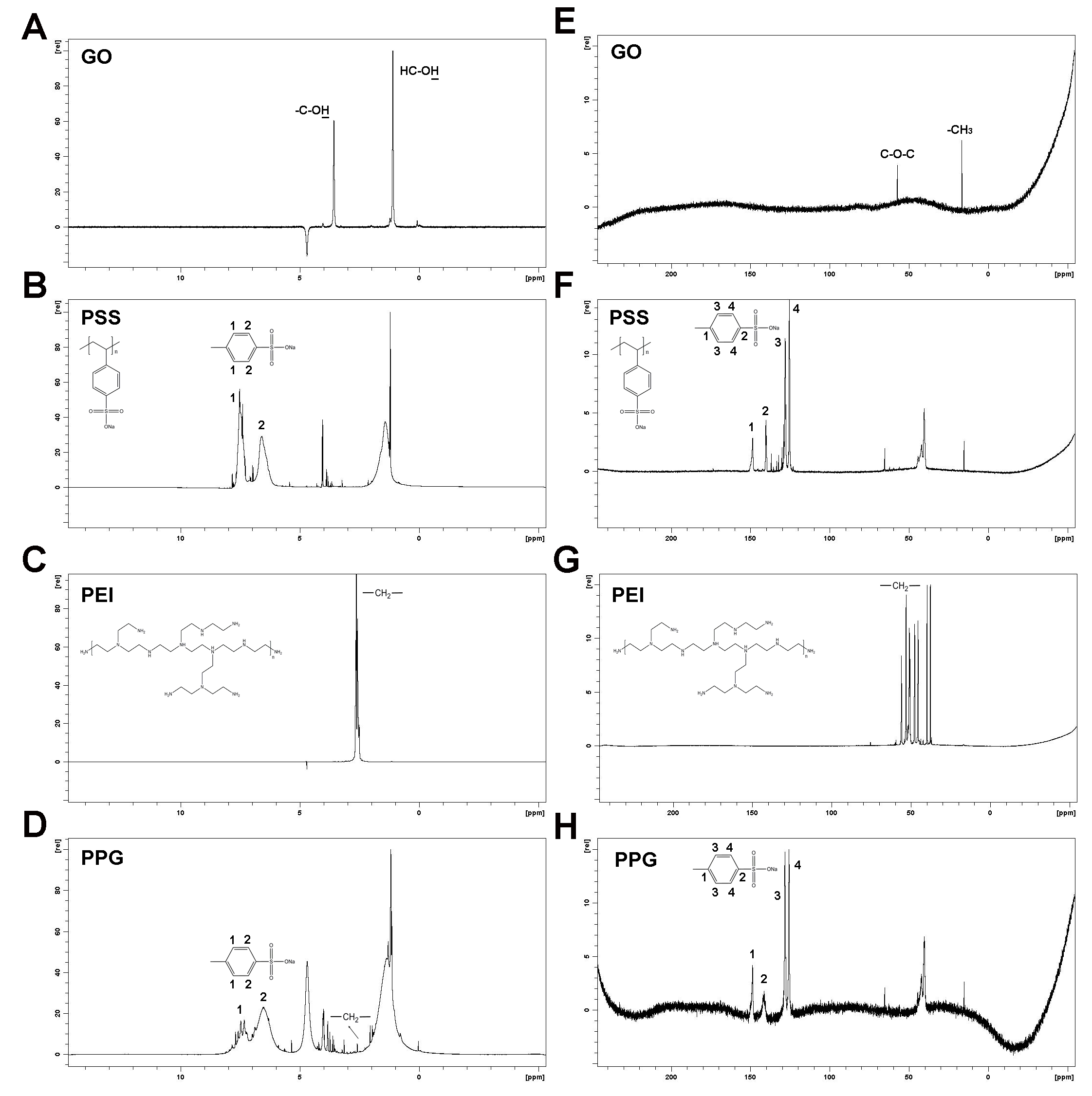

Supplement: Figure S2 — Characterization of PPG by 1H and 13C MAS NMR spectra. Liquid-state 1H MAS NMR spectra of GO (A), PSS (B), PEI (C) and PPG (D), Liquid-state 13C MAS NMR spectra of GO (E), PSS (F), PEI (G) and PPG (H). (TIF) [file pone.0060034.s002.tif]
